# Over-the-counter emergency contraception in Italy: ethical reflections and medico-legal issues

**DOI:** 10.3389/fgwh.2023.1205208

**Published:** 2023-09-18

**Authors:** Roberto Scendoni, Mariano Cingolani, Fabio Cembriani, Piergiorgio Fedeli, Vittoradolfo Tambone, Corrado Terranova, Francesco De Micco

**Affiliations:** ^1^ Department of Law, Institute of Legal Medicine, University of Macerata, Macerata, Italy; ^2^ Operative Unit of Legal Medicine, Provincial Authority for Health Services of Trento, Trento, Italy; ^3^ School of Law, Legal Medicine, University of Camerino, Camerino, Italy; ^4^ Fondazione Policlinico Universitario Campus Bio-Medico, Roma, Italy; ^5^ Research Unit of Bioethics and Humanities, Department of Medicine and Surgery, Università Campus Bio-Medico di Roma, Roma, Italy; ^6^ Research Unit of Gynaecology, Department of Medicine and Surgery, Università Campus Bio-Medico di Roma, Roma, Italy

**Keywords:** ulipristal acetate, emergency contraception, human reproduction, informed consent, medical ethics

## Abstract

Although more than ten years have passed since the marketing of Ulipristal acetate in Europe, emergency contraception remains a complex issue with many scientific, legal, ethical and social implications. The topic is an example of the differences that can exist between scientific evidence, the certainties on which law is based, and social implications. This paper shows the incompleteness of the scientific reconstruction on the effects of emergency hormonal contraceptives and the dangerousness of the decision to alienate the supply of over-the-counter drugs from the general rules of health care. This report shows the incompleteness of the scientific reconstruction on the effects of emergency hormonal contraceptives and the dangerousness of the decision to alienate the supply of over-the-counter drugs from the general rules of health care. Various ethical and medico-legal issues will be addressed, also focusing attention on underage women whose sexual and reproductive health requires not abandoning them, but actually taking charge of them without medicalizing their choices.

## 1. Introduction

Emergency contraception (EC) refers to methods of contraception that can be used to prevent pregnancy after sexual intercourse. These are recommended for use within 5 days but are more effective the sooner they are used after the act of intercourse (1).

This definition depends on the meaning of the word conception, which was defined by the American College of Obstetricians and Gynecologists in 1965 as the implantation of a fertilized egg in the uterus (2).

According to Scotson, there is a terminological misunderstanding: if taking the postcoital contraceptive pill serves to prevent implantation of a fertilized egg, thus blocking any further development, does this in effect amount to a chemical abortion (3)? If so, the term EC is erroneous and we should perhaps instead refer to emergency interception or emergency pregnancy termination (4).

In fact, scientists disagree over how the pills actually work. There is no consensus about the effects of synthetic progestins (levonorgestrel or LNG) and selective progesterone modulators (ulipristal acetate). For some, these drugs simply delay or inhibit ovulation (and therefore block the creation of fertilized eggs), but for others, they can act after fertilization (5–8).

The possibility that these drugs could prevent a fertilized ovum from implanting in the mother's uterus has raised complex questions about the woman's freedom of choice, the protection of the unborn child, and the right of health professionals to refrain for conscientious reasons from prescribing and dispensing such drugs to the public, even when a regular prescription is presented (6).

The ethical and medico-legal issues are so significant that the Italian Committee for Bioethics, when called upon to express an opinion on EC, made two points: on the one hand, there were those who considered the pre-fertilization effect of LNG to be prevalent or exclusive; on the other hand, the concrete possibility of the drug having post-fertilization effects was highlighted. Therefore, it was unanimously considered lawful for a physician to invoke conscientious objection and refuse to prescribe or dispense LNG (9).

With regard to pharmacists’ conscientious objections to the sale of emergency contraceptives, different positions emerged within the Committee. Some members recognized the pharmacist as sharing the same role that can be attributed to other health care workers and therefore as having the same right to conscientious objection. Other members did not consider the pharmacist to be on the same footing as the medical doctor: the pharmacist, then, may not override the physician's decision nor interfere in the woman's private life (10).

Alongside the ethical issues, EC has many bio-legal implications. The last act in what has been a long judicial drama was the Italian Council of State's decision to confirm the legitimacy of AIFA's resolution to withdraw the prescription requirement for ulipristal acetate for minors (11).

According to the Italian Council of State, over-the-counter drugs that do not require a medical prescription, such as those used for EC, should not be considered medical treatment. Therefore, personal, free, explicit, informed, specific, current, and revocable consent at any time is not required. Moreover, in the case of EC, the need for parental or guardian consent would expose the minor to violation of her sexual freedom and privacy. Lastly, if the mechanism of action (MOA) of ulipristal acetate is anti-ovulatory, taking the pill would not incur a breach of the law on voluntary termination of pregnancy (11).

This latest judgment has not solved the matter but provides a starting point for new avenues of thought.

The most relevant ethical and medico-legal aspects at stake concern the post-fertilization and abortifacient effects of emergency contraception, the lawfulness of conscientious objection invoked by the medical doctor and pharmacist, and the correctness of information regarding drug prescriptions.

The aim of this article is to renew the discussion on the difficult relationship that exists between science and law in situations where scientific evidence is not yet sufficient to withstand falsifiability checks (12).

## 2. Emergency contraception: the doubts of science, the certainties of law

Since the Italian Medicines Agency granted approval for marketing EC, the scientific, legal, and ethical debates surrounding the issue have been very heated.

Immediately after the launch of NorLevo, some pro-life organizations filed an appeal with the Regional Administrative Court of Lazio in an effort to invalidate the Ministry of Health's decree authorising the marketing of LNG. Their fundamental argument was that the product information included in the package insert was not enough to allow women to qualify the drug's mechanism of action (MOA) as either contraceptive or abortifacient (13), an important distinction depending on their ethical and religious orientation.

The Regional Administrative Court of Lazio partially upheld the appeal, considering it necessary to provide full and detailed information regarding the possible abortifacient activity of the drug in view of the different ethical and religious perspectives on the beginning of human life, so as to make it clear and unequivocal that the drug has the potential to act on the already fertilized ovum, preventing subsequent phases of the biological process of procreation (14).

In 2004, the National Bioethics Committee of Italy gave physicians the option of invoking conscientious objection to refuse the prescription or administration of LNG due to possible post-fertilization effects (9).

In 2015, the Italian Superior Health Council argued that minors should only be able to purchase ulipristal acetate in a pharmacy with a medical prescription (15), on the grounds that the non-prescription marketing of ulipristal acetate can result in decreased use of hormonal and mechanical contraceptives which are useful in the prevention of sexually transmitted diseases (STDs). The Council highlighted the main reason why the European Medicines Agency (EMA) authorised non-prescription marketing in the first place: removing the need to obtain a prescription from the physician was seen as a way of speeding up access to this medicine, thus increasing its effectiveness. However, for the Italian Superior Council, this consideration did not apply to the Italian scenario where the availability of medical counselling is guaranteed free of charge and on a continuous basis (15).

However, the Italian Medicines Agency cancelled the prescription requirement for the marketing and dispensing of ulipristal acetate to women over the age of 18 (16).

The choice to make medical prescriptions obligatory for the dispensing of LNG in Italy contrasts with decisions taken by other European countries (with the exception of Hungary) and non-European countries (e.g., the State of California, Hawaii, and Washington) where LNG is considered an over-the-counter product that can be dispensed without a medical prescription (17).

In 2020, the Italian Medicines Agency ruled that a medical prescription was no longer required to dispense ulipristral acetate to females under the age of 18 (18).

The Council of State rejected the appeal of pro-life associations against the Italian Medicines Agency's decision arguing that ulipristal acetate's MOA is anti-ovulatory, acting before implantation of the embryo (11). The Italian Medicines Agency supported its decision by reporting that the safety and quality of ulipristal acetate were ensured in a sample of girls over the age of thirteen and in a sample of adult women over the age of eighteen.

Moreover, the same judges declared that the previous opinions of the Higher Health Council of 2015 were irrelevant, as well as scientific studies conducted in the early 2000s.

In summary, the Council of State held that the scientific literature unanimously denied the antinidatory effect of ulipristal acetate. However, this conclusion was opposed by the scientific evidence that ulipristal acetate could modify the uterine environment by altering the mechanism that leads to decidualization of the endometrium in response to progesterone (19, 20).

It may be argued that this evidence needs further confirmation, but it is reasonable to point out that scientific doubts about the effect of ulipristal acetate still exist despite the fact that its main action is to prevent or delay ovulation. Therefore, the categorical conclusions reached by the judges may come as a surprise; they should have acknowledged that the matter is much more complex because the scientific evidence is neither complete nor definitive.

These considerations also apply to LNG, which requires the presentation of a medical prescription in order to be taken by a minor. Pharmacokinetic and pharmacodynamic studies have shown that LNG prevents pregnancy with a mechanism of action similar to other hormonal contraceptives by preventing or delaying ovulation when taken by women in the pre-ovulatory phase (21). In vitro, LNG has been shown to alter the characteristics of cervical mucus (22), interfere with sperm motility (23), modify tubal motility (24), and prevent implantation of the fertilized egg in the mother's uterus by altering the physiology and function of the endometrium, acting both at the level of the so-called implantation factors and locally (25, 26).

In vitro, LNG acts on certain target organs: the fallopian tubes, the cervix, the endometrium, and even the breasts. These actions bring about a plurality of effects that can be traced back to: (a) the interference exerted on ovulation that can be inhibited and/or delayed; (b) the possibility of a post-fertilization action (contragestative or abortive) through modification of the uterine mucosa, alteration of tubal motility, the effect on the endometrium, and the effect on implantation factors in the event that fertilization has actually occurred.

These latter effects are still a matter of scientific debate. Some studies have shown that LNG does not modify the endometrium (27), that ectopic pregnancies are rare (28), and that the drug, in addition to interfering with ovulation, is able to prevent the spermatozoa from coming into contact with the oocyte, thus discounting clinical data supporting mechanisms other than ovulation inhibition, delay, or impairment (29).

The methodology of the latter study has been criticized: the ICEC/FIGO conclusions were derived from a review of just seven studies involving a total of only 142 patients which were further divided into several subgroups, making statistically significant conclusions impossible (30). One of the largest studies conducted on Plan B shows that it can delay ovulation when taken before or at the beginning of the fertile period, when it is not necessary to prevent pregnancy; when the drug is given after intercourse in the fertile period and before the LH surge that induces ovulation, Plan B fails as a contraceptive 80%–92% of the time and instead acts as an abortifacient, eliminating all embryos that are likely to have been conceived (31, 32).

The potential for LNG to have an abortive effect is by no means negligible, and therefore it is not reasonable to deny that it has post-fertilization effects (33, 34).

## 3. The ethical and legal issue of minors

Particular attention should be paid to the category of minors. Without a medical prescription, an adolescent might approach a pharmacist to ask for ulipristal acetate even though they are not yet fully capable of acting and self-determining in the field of their sexual and reproductive health. This could be perceived as potentially conflicting with the Italian law on informed consent and advance treatment directives: the right of minors to receive full and correct information (since reading only the product leaflet is not enough for this purpose); and the right and duty of parents or guardians to protect their child's health while considering the child's wishes in relation to age and maturity (35). Differences of opinion and clashes with one or more parents can easily arise. In this case, it would be advisable to make every effort to facilitate an agreement between the minor (endowed with capacity for discernment) and the parents, to guide the entire family nucleus towards the best choice for the minor, both from a clinical health point of view and from an ethical and social point of view (36, 37).

The judges of the Council of State addressed this critical issue by arguing that drugs that can be dispensed without a prescription are not comparable to health treatments that require the patient's informed consent and the physician-patient relationship (38). Indeed, the issue is complicated and deserves more attention. Cognitive biases can originate from erroneous prejudices with dangerous practical effects. According to the Italian penal code, a 13-year-old female cannot sexually dispose of her body, which could imply that dispensing pharmacological contraception amounts to facilitating illicit sexual behavior.

Among other things, the issue of voluntary interruption of pregnancy merits further consideration in relation to Italian law. If the medication effect of EC is of the contraceptive type, the provisions are as set forth in Article 2 of the Law on Voluntary Termination of Pregnancy; if the effect of drugs used for EC is abortifacient, then Article 9 of the same law should be considered (35). According to Article 9, conscientious objection exempts health care workers from carrying out procedures and activities specifically and necessarily aimed at bringing about the abortion of pregnancy. Article 2 of the same law made public health facilities responsible for ensuring that the reproductive protection of minors is never addressed only pharmacologically. Indeed, a diligent and prudent multidisciplinary (medical, obstetrical, psychological) intervention is required to assess the minor's psychological maturity.

Moreover, a point that has not yet been raised is the key role of the physician in providing information on sexuality, reproduction, and contraception. According to the Italian Code of Ethics, the doctor must provide individuals and couples with all the necessary information on sexuality, reproduction, and contraception in order to protect individual and collective health and conscious and responsible procreation (39).

## 4. Conclusion

EC remains an extraordinarily complex issue due to its biological, ethical, and legal implications and social consequences.

Among these, the issue of conscientious objection raises significant questions concerning the moment of the beginning of life. Indeed, the possibility of contravening obligations imposed by law because of ethical and religious principles rooted in the intimate sphere of the objector is an issue that still divides society (40–42).

The European Court of Human Rights (ECHR) has repeatedly addressed this issue, trying to balance the rights of medical doctors and health care workers with those of patients.

The Court ruled that conscientious objection must be legitimised by sincere and strongly held convictions and patients must be informed about their treatment options, including the alternatives available. In addition, healthcare facilities must ensure that patients have access to the care they need without undue discrimination (43).

Italian jurisprudence also made its own contribution to the debate concerning the balancing of rights in the area of conscientious objection. According to the Italian Constitutional Court, the protection of individual conscience receives constitutional protection in line with the need for those freedoms and rights not to be unreasonably compressed. Therefore, the juridical potential of the individual conscience is a constitutional value so high as to justify the provision of privileged exemptions from the performance of public duties qualified as non-derogable (44).

After the marketing authorization of ulipristral acetate, the issue raised wide-ranging questions that the Italian courts have attempted to address.

In this research, we have examined the judgments of the Regional Administrative Court of Lazio and the Italian Council of State resulting from the legal appeals lodged by pro-life associations against the decision of the Director General of the Italian Medicines Agency to authorize the sale of LNG.

The analyzed resolutions have failed to convince us, since they raise important critical issues that remain unanswered. First of all, the judgment did not consider the scientific evidence proving the abortive effect of ulipristal acetate. Secondly, the judgments did not address the critical issues that arise when ulipristal acetate is requested by a minor without any medical supervision, in some cases even unaccompanied (45, 46). It is simplistic to reduce the issue in this way, and nothing is solved by separating the woman's choice from rules guaranteeing health treatment.

Stating that a woman's reproductive choice has nothing to do with her health and the legislative protection of health is a very dangerous idea: it could justify the danger of a “do-it-yourself” approach, which may prevent minors from facing core problems surrounding their sexuality in certain places. A woman's reproductive choice and responsible motherhood are not addressed by hormonal contraception alone. A “double contraceptive pathway” is created by forcing the woman to go to the specialist physician for precoital oestrogen contraception and then leaving her to seek emergency postcoital contraception directly at a pharmacy that dispenses the drug without any medical supervision.

As well as being unreasonable, the path outlined is dangerous and needs serious reflection on it, focusing not only on informed consent but also on the protection of significant principles: the protection of health as a fundamental right of the individual and an interest of the community and solidarity as a social principle and moral virtue. Especially when the principle of solidarity concerns the most fragile and vulnerable people who must never be abandoned to their fate by liberalising their life choices, especially when they require the possession of a good degree of maturity, even if the path of medicalisation is not the solution that can calm all our anxieties. It is not a question of medicalising the person's life choices concerning his or her alute but of offering him or her the necessary support whenever his or her self-determination is in danger of being put on the back burner by contingent factors.

## Data Availability

The original contributions presented in the study are included in the article/Supplementary Material, further inquiries can be directed to the corresponding author.
